# Supplementary material for: Droughts worsen air quality and health by shifting power generation
Source: Nat Commun. 2025 May 22;16:4774. doi: 10.1038/s41467-025-60090-z (PMC12098700; doi:10.1038/s41467-025-60090-z)
Supplement: Supplementary file 1 — Supplementary Information [file 41467_2025_60090_MOESM1_ESM.pdf]

Droughts Worsen Air Quality and Health by Shifting Power Generation

Supplementary Information

Mathilda Eriksson[1*], Alejandro del Valle[2*] and Alejandro de la Fuente[3]

[1]Maurice R. Greenberg School of Risk Sciences, Georgia State University, 35 Broad Street NW, Atlanta, 30303, GA, USA.
[2]Maurice R. Greenberg School of Risk Sciences, Georgia State University, 35 Broad Street NW, Atlanta, 30303, GA, USA.
[3]Poverty and Equity Global Practice, World Bank Group, Delta Center, Menengai Road, Upper Hill, Nairobi, Kenya.

*Corresponding authors. E-mail: meriksson@gsu.edu; adelvalle@gsu.edu;

Supplementary Items List

Supplementary Figures 1–14

Supplementary Tables 1–2

Supplementary Methods 1–2

Supplementary References

Supplementary Figures

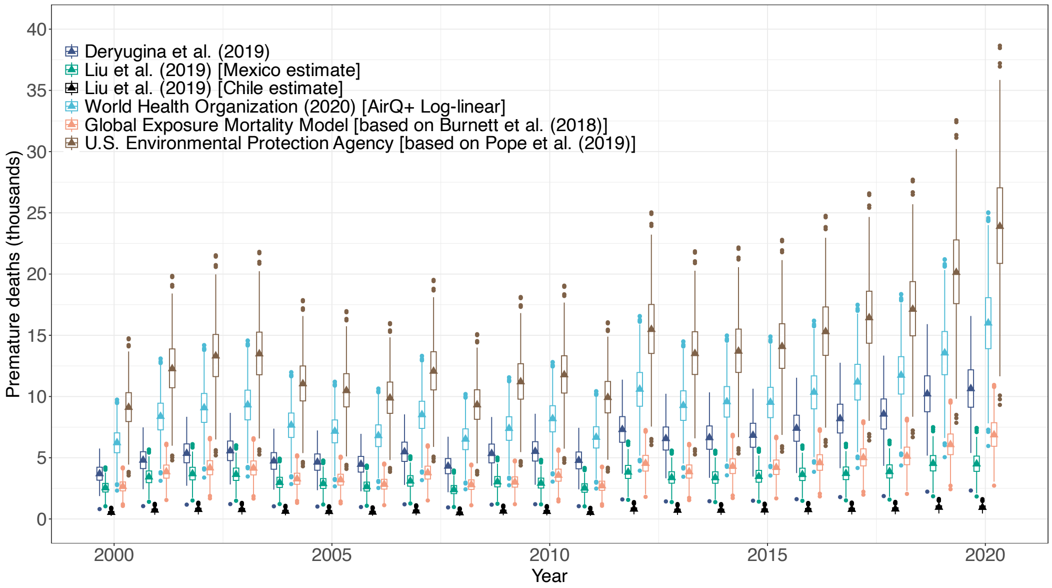

Supplementary Figure 1 Premature deaths from drought-induced excess $PM_{2.5}$ using alternative concentration-response functions. See Supplementary Methods for details of the calculation. LAC-level annual time series of premature deaths (N = 21,000 simulation-year units; 1,000 draws × 21 years). The spread of the box plots displayed each year results from the uncertainty in our estimation of excess $PM_{2.5}$ concentrations and the value of the observed FHD. Box plots indicate median (middle line), 25th, 75th percentile (box), 1.5 times the interquartile range (whiskers), outliers (single points), and mean values (triangles). Source data are provided as a Source Data file (sourcedata.xlsx). The data and code used to obtain the estimates are available at https://www.openicpsr.org/openicpsr/project/217201.

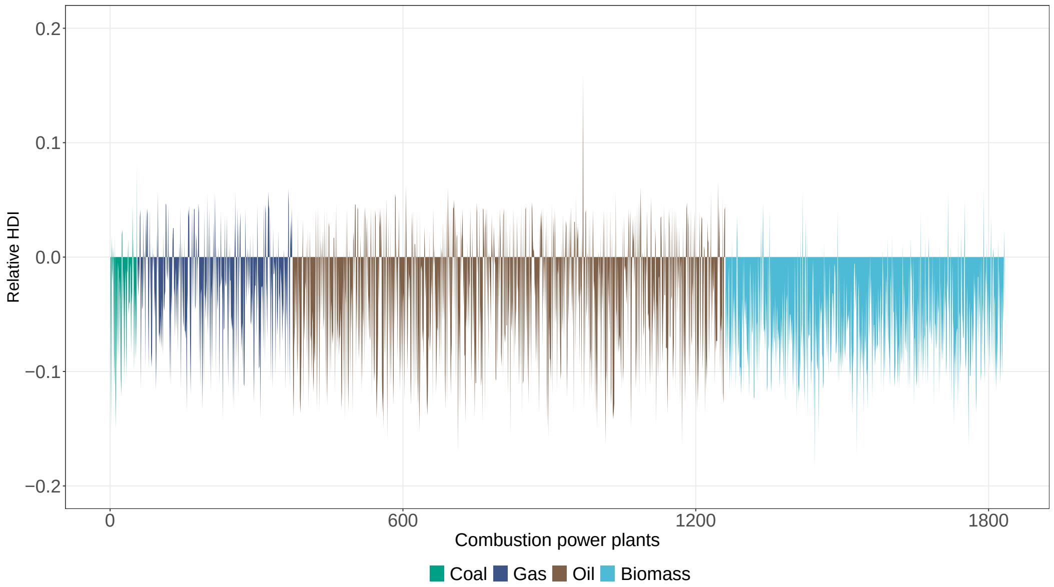

Supplementary Figure 2 Combustion power plant HDI relative to country HDI. This figure plots for each combustion power plant the difference between the average Human Development Index (HDI) among the population residing within 50 km and the HDI of the country where the plant is located (N = 1,835). The HDI data corresponds to 2019. Source data are provided as a Source Data file (sourcedata.xlsx). The data and code used to obtain the estimates are available at https://www.openicpsr.org/openicpsr/project/217201.

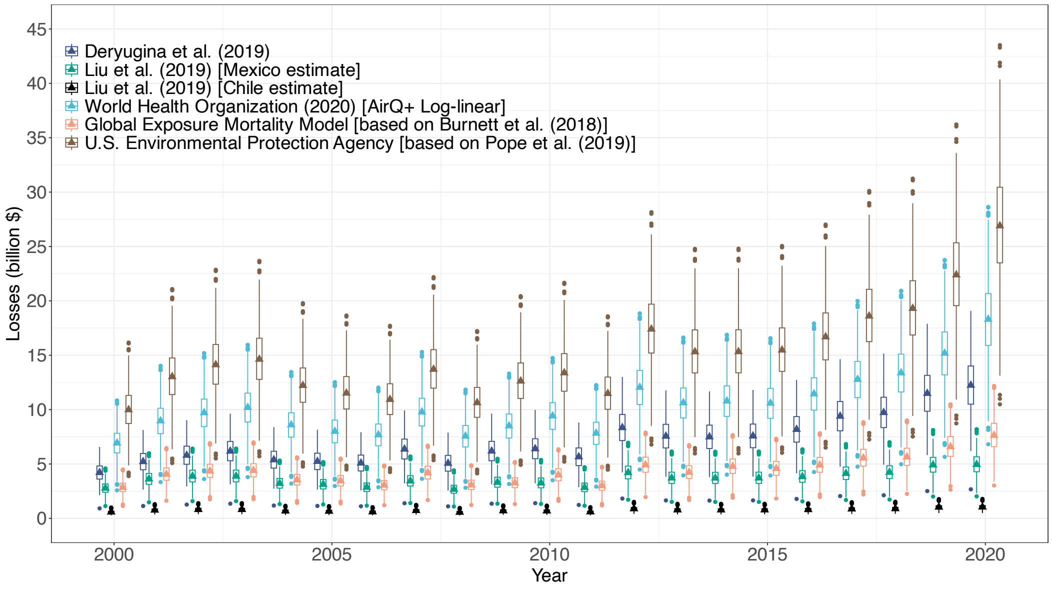

Supplementary Figure 3 Monetized losses from drought-induced excess $PM_{2.5}$ using alternative premature death estimations. Premature deaths from Fig. 1 are monetized to 2019 USD using country-year estimates of the value of a statistical life extrapolated from US estimates. LAC-level annual time series of losses (N = 21,000 simulation-year units; 1,000 draws × 21 years). The spread of the box plot displayed each year results from the uncertainty in our estimation of excess $PM_{2.5}$ concentrations and the value of the observed FHD. Box plots indicate median (middle line), 25th, 75th percentile (box), 1.5 times the interquartile range (whiskers), outliers (single points), and mean values (triangles). Source data are provided as a Source Data file (sourcedata.xlsx). The data and code used to obtain the estimates are available at https://www.openicpsr.org/openicpsr/project/217201.

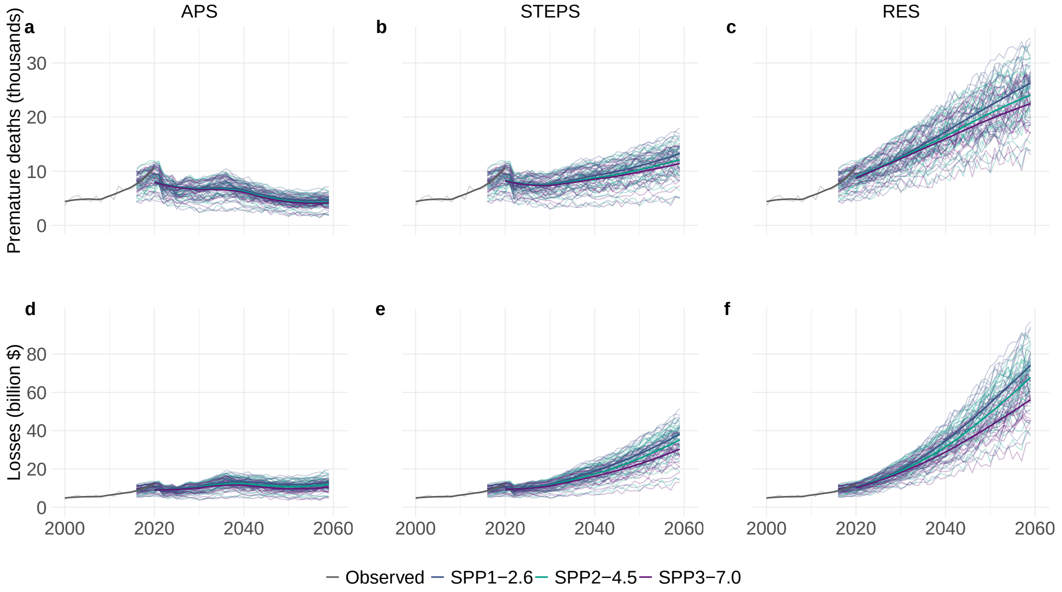

Supplementary Figure 4 Projected premature deaths and losses from drought-induced excess $PM_{2.5}$ under SSP-RCP and energy policy scenarios. a–f Observed and projected premature deaths (thousands, a-c) and losses (billion 2019 USD, d-f) from 2000 to 2059 under three energy policy pathways: Announced Pledges Scenario (APS, a,d), Stated Policies Scenario (STEPS, b,e), and Reference Electricity Scenario (RES, c,f). Estimates are based on (i) runoff projections from 22 climate and earth system models under three Shared Socioeconomic Pathways and Representative Concentration Pathways (SSP-RCP) scenarios—SSP1-2.6, SSP2-4.5, and SSP3-7.0; (ii) demographic and economic growth projections aligned with the same SSP-RCP scenarios; and (iii) combustion power plant retirement schedules under the energy policy pathways from the IEA [1]. The APS assumes retirements in line with country pledges, the STEPS reflects the IEA's assessment of the region's energy direction, and the RES assumes no retirements. All projections assume that additional electricity demand is met with non-combustion power, no new combustion power plants are introduced, and the concentration-response function remains constant (no adaptation). Bold lines represent LOESS curve fits. Source data are provided as a Source Data file (sourcedata.xlsx). The data and code used to obtain the estimates are available at https://www.openicpsr.org/openicpsr/project/217201.

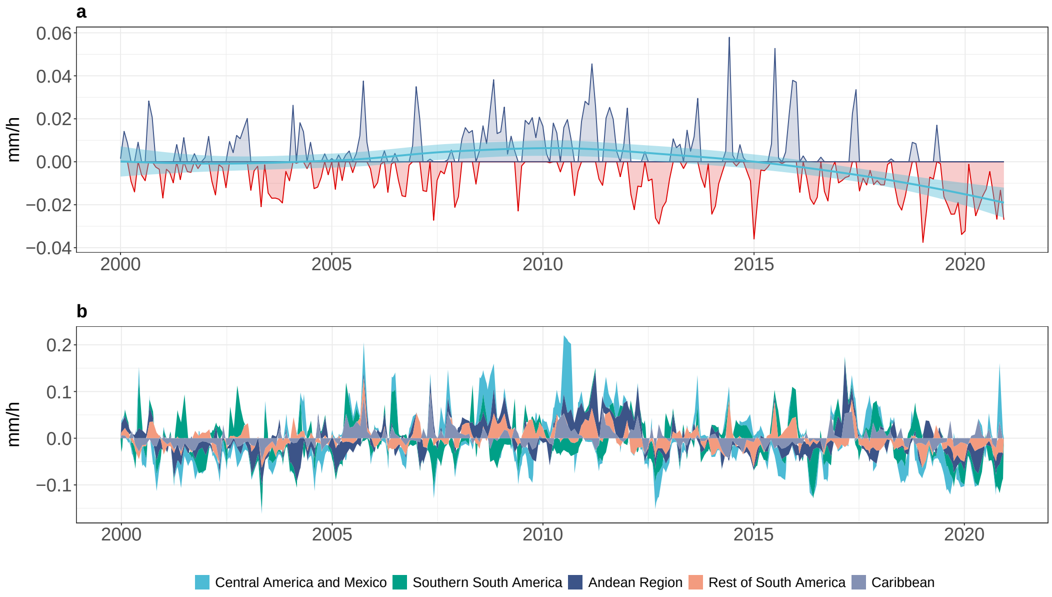

Supplementary Figure 5 Runoff anomalies in hydropower watersheds over time. a LAC level mean monthly runoff anomalies based on N = 269,388 month-watershed observations (12 months × 21 years × 1,069 watersheds). The blue-shaded areas represent positive runoff anomalies. The red-shaded areas represent negative runoff anomalies. The LOESS curve visualizes the trend over this period. **b** Mean monthly runoff anomalies by IEA sub-region. The sub-regions are: Central America and Mexico, Southern South America (Argentina, Bolivia, and Chile), Andean Region (Colombia, Ecuador, and Peru), Rest of South America (Brazil, Venezuela, Paraguay, and Uruguay), and Caribbean. Source data are provided as a Source Data file (sourcedata.xlsx). The data and code used to obtain the estimates are available at https://www.openicpsr.org/openicpsr/project/217201.

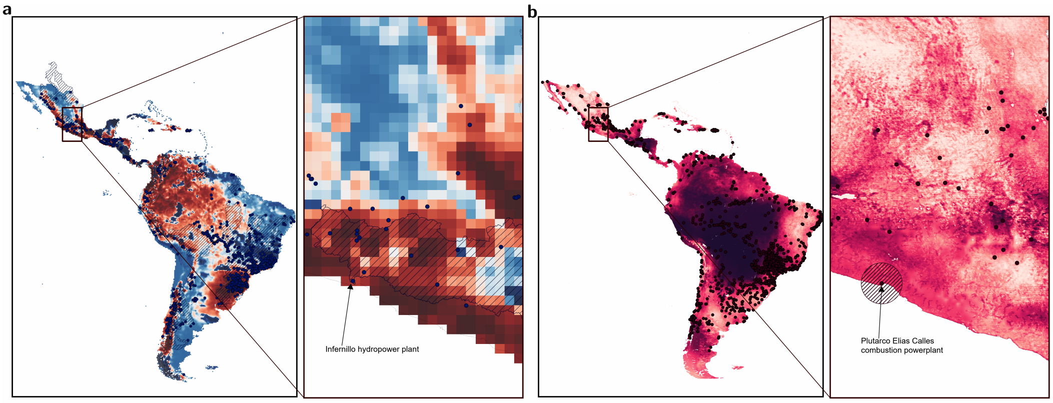

Supplementary Figure 6 Distribution of power plants, watersheds, hydrological drought, and air pollution. a Blue dots represent hydropower plants (N = 1,069), hatched areas show hydropower watersheds, and the color gradient indicates the average monthly runoff anomalies ranging from low (red) to high (blue). Data corresponds to October 2020. The zoomed panel shows the Mexican hydropower plant Infernillo and its watershed. **b** Red dots represent combustion power plants (N = 1,835), hatched circles show the 50 km radius around combustion power plants, and the color gradient indicates $PM_{2.5}$ concentrations ranging from low (light purple) to high (dark purple). Data corresponds to October 2020. The zoomed panel shows the Mexican coal power plant Plutarco Elías Calles. Administrative boundary data were obtained from the Database of Global Administrative Areas (GADM), version 4.1, available at www.gadm.org.

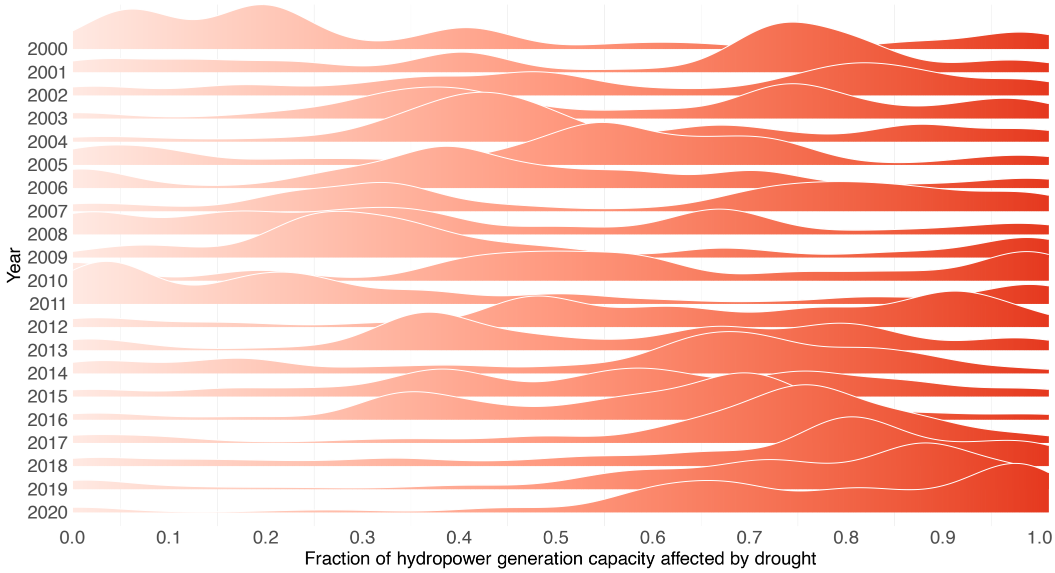

Supplementary Figure 7 Annual distribution of FHD values. The ridgeline plot shows the annual distribution of the fraction of hydropower generation affected by drought (FHD) over the 20-year study period (N = 80,355 plant-month observations). Each ridge represents the density of FHD values for a specific year, with shading from light red (FHD = 0, no hydropower generation affected by drought) to dark red (FHD = 1, all hydropower generation affected by drought). Source data are provided as a Source Data file (sourcedata.xlsx). The data and code used to obtain the estimates are available at https://www.openicpsr.org/openicpsr/project/217201.

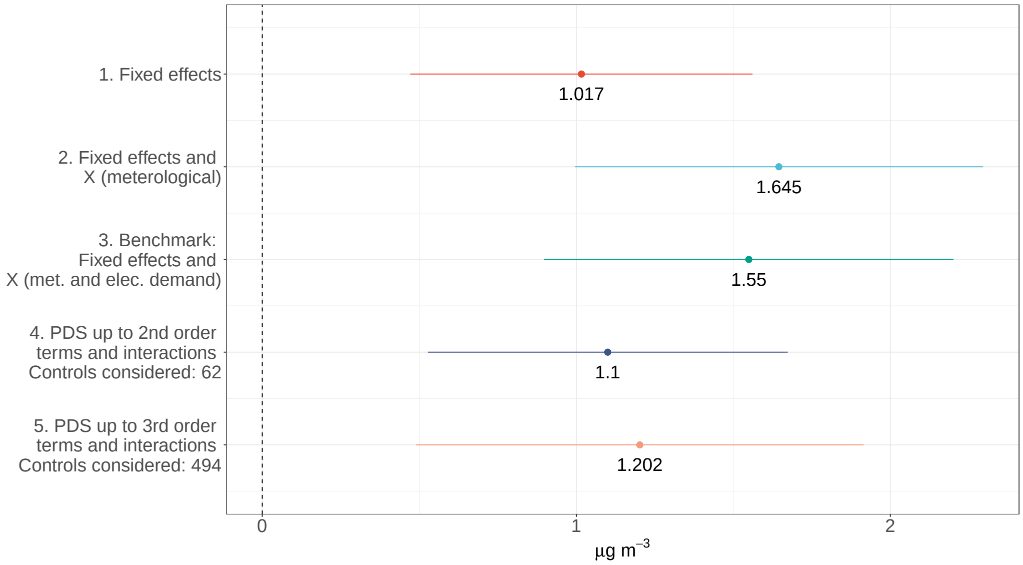

Supplementary Figure 8 Robustness of estimates to alternative model specifications. All results are based on the analysis sample (N = 79,022 plant-month observations). The figure plots point estimates (points) and 95% confidence intervals (error bars) of the β coefficient from five specifications of Equation (1). The CIs are derived from standard errors clustered at the market level (19 clusters). All specifications use FHD as our preferred measure of hydrological drought (HD). Models 1 to 3 estimate specification Equation (1) using OLS but introduce the controls in steps as described in the axis title. In models 4 and 5, we estimate Equation (1) using the post-double selection method of Belloni et al.[2]. The model specification and number of controls considered are specified in the axis title. Source data are provided as a Source Data file (sourcedata.xlsx). The data and code used to obtain the estimates are available at https://www.openicpsr.org/openicpsr/project/217201.

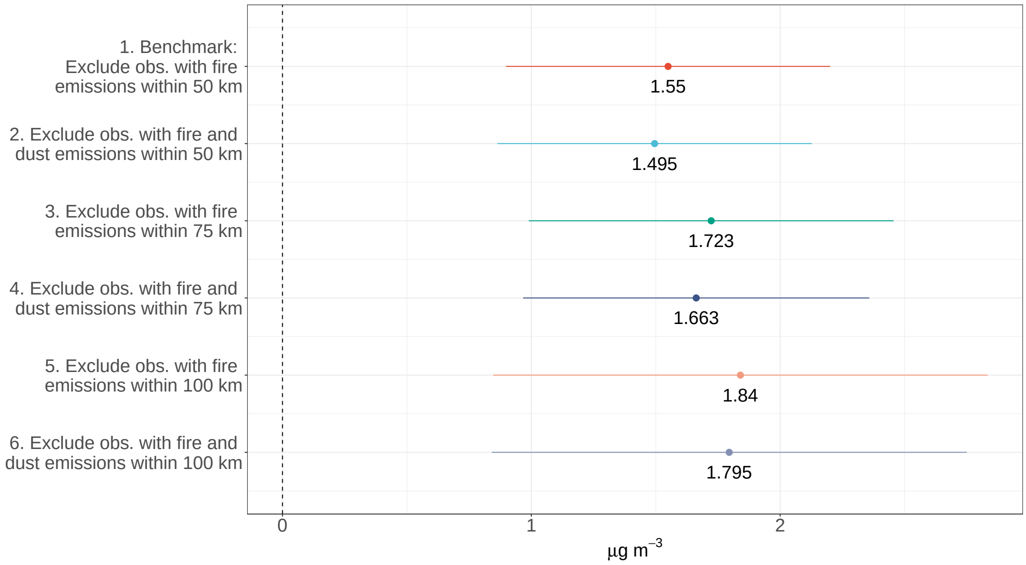

Supplementary Figure 9 Robustness of estimates to exclusion of plant-month observations potentially affected by fires or dust storms. The figure plots point estimates (points) and 95% confidence intervals (error bars) of the β coefficient from six specifications of Equation (1). The CIs are derived from standard errors clustered at the market level (19 clusters). All specifications use FHD as our preferred measure of hydrological drought (HD). Each specification excludes the observations indicated in the corresponding row title. The sample sizes are as follows: model 1 (N = 79,022 plant-month observations), model 2 (N = 74,998), model 3 (N = 50,263), model 4 (N = 46,848), model 5 (N = 36,687), model 6 (N = 33,643). Source data are provided as a Source Data file (sourcedata.xlsx). The data and code used to obtain the estimates are available at https://www.openicpsr.org/openicpsr/project/217201.

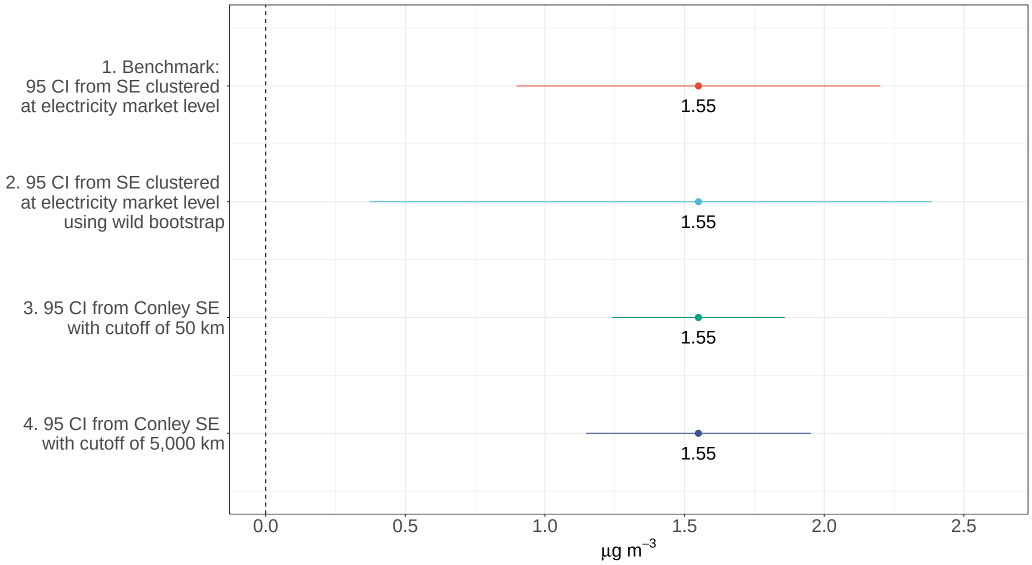

Supplementary Figure 10 Robustness of estimates to standard errors under alternative assumptions. All results are based on the analysis sample (N = 79,022 plant-month observations). The figure plots point estimates (points) and 95% confidence intervals (error bars) of the β coefficient from four specifications of Equation (1). All specifications use FHD as our preferred measure of hydrological drought (HD). The confidence intervals are derived from four types of standard errors. Model 1 uses standard errors clustered at the market level (19 clusters). Model 2 uses standard errors clustered at the market level (19 clusters) but accounts for few clusters using the wild bootstrap methodology (see Roodman et al.[3] for details of the calculation). Models 3 and 4 use Conley errors that assume all available lags and the cut-off described in the axis title (see Colella et al.[4] for details of the calculation).Source data are provided as a Source Data file (sourcedata.xlsx). The data and code used to obtain the estimates are available at https://www.openicpsr.org/openicpsr/project/217201.

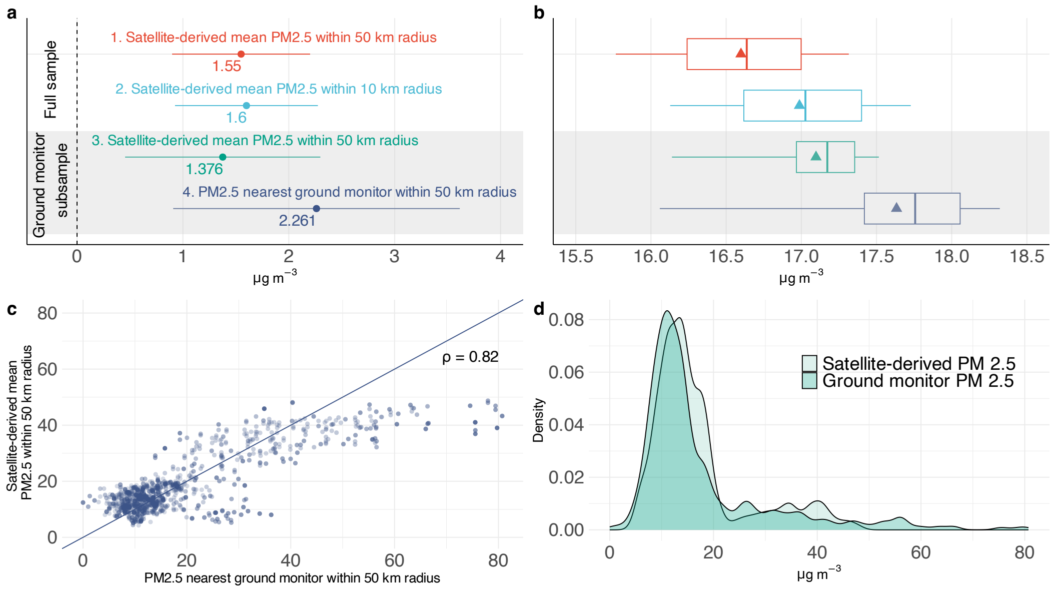

Supplementary Figure 11 Robustness of estimates under alternative $PM_{2.5}$ measures and validation of satellite-derived $PM_{2.5}$ against ground-based measurements. a The figure plots point estimates (points) and 95% confidence intervals (error bars) of the β coefficient from four specifications of Equation (1). The CIs are derived from standard errors clustered at the market level. The full sample corresponds to the analysis sample (N = 79,022). For this sample, the outcomes are mean monthly satellite-derived $PM_{2.5}$ concentrations measured within 50 km and 10 km of a combustion power plant. The ground monitor subsample (N = 1,797) comprises plant-month observations with available ground monitor data within a 50 km radius. For this subsample, the outcomes are satellite-derived $PM_{2.5}$ concentrations within a 50 km radius and $PM_{2.5}$ measurements from the nearest ground monitor within 50 km. **b** Distribution of implied total $PM_{2.5}$ concentrations, calculated as the marginal effect plus the predicted $PM_{2.5}$ level in the absence of droughts. Box plots indicate median (middle line), 25th, 75th percentile (box) and minimum and maximum (whiskers) as well as mean values (triangles). **c** Scatter plot of satellite-derived mean monthly $PM_{2.5}$ concentrations within 50 km versus observed monthly $PM_{2.5}$ from nearest ground monitoring station within 50 km. The Pearson correlation (ρ) is reported in the top right corner (N = 1,797). **d** Kernel density plots comparing the distributions of satellite-derived and ground monitor $PM_{2.5}$ concentrations (N = 1,797). Source data are provided as a Source Data file (sourcedata.xlsx). The data and code used to obtain the estimates are available at https://www.openicpsr.org/openicpsr/project/217201.

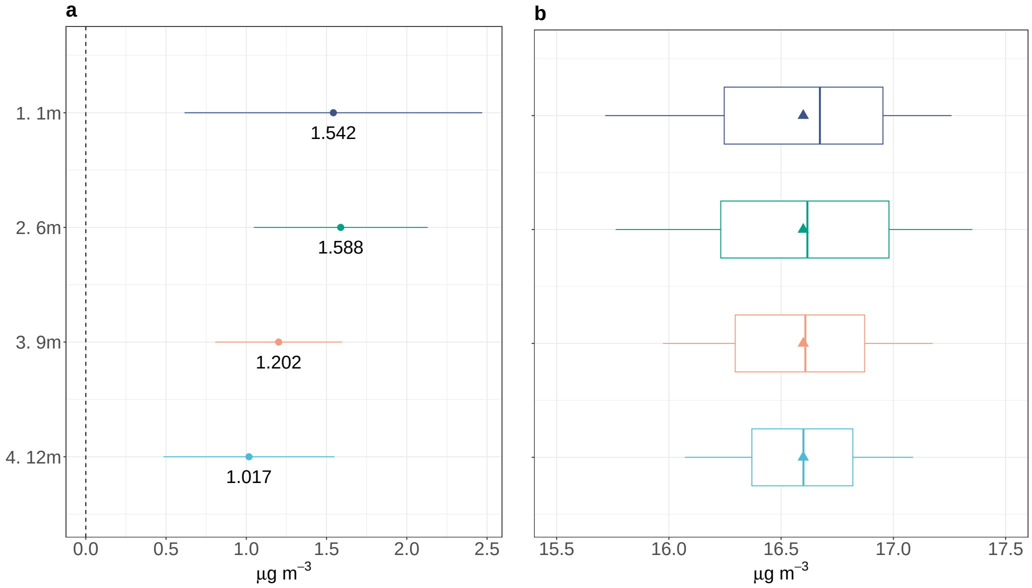

Supplementary Figure 12 Effect of FHD on $PM_{2.5}$ concentrations under alternative definitions of drought duration. All results are based on the analysis sample (N = 79,022 plant-month observations). **a** The figure plots point estimates (points) and 95% confidence intervals (error bars) of the β coefficient from four specifications of Equation (1). The CIs are derived from standard errors clustered at the market level (19 clusters). All specifications use FHD as the measure of hydrological drought (HD). Each model uses a version of the FHD derived under the definition of drought listed in the axis title (1, 6, 9, and 12 months). **b** Distribution of implied total $PM_{2.5}$ concentrations, that is, the marginal effect plus the predicted level of $PM_{2.5}$ in the absence of droughts. Box plots indicate median (middle line), 25th, 75th percentile (box), and minimum and maximum (whiskers) as well as mean values (triangles). Source data are provided as a Source Data file (sourcedata.xlsx). The data and code used to obtain the estimates are available at https://www.openicpsr.org/openicpsr/project/217201.

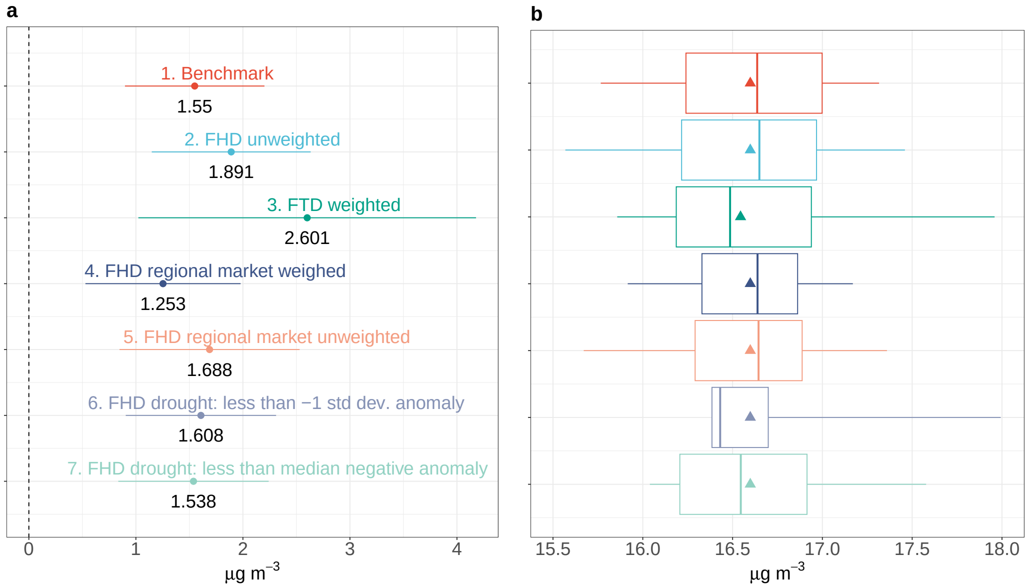

Supplementary Figure 13 Robustness of estimates under alternative definitions of treatment. All results are based on the analysis sample (N = 79,022 plant-month observations). **a** The figure plots point estimates (points) and 95% confidence intervals (error bars) of the β coefficient from seven specifications of Equation (1). The CIs are derived from standard errors clustered at the market level. Models 1 to 7 report estimates from Equation (1), each using the market-level measure of hydropower generation affected by drought listed on top of the coefficient. **b** Distribution of implied total $PM_{2.5}$ concentrations, i.e., the marginal effect plus the predicted level of $PM_{2.5}$ in the absence of droughts. Box plots indicate median (middle line), 25th, 75th percentile (box), and minimum and maximum (whiskers) as well as mean values (triangles). Source data are provided as a Source Data file (sourcedata.xlsx). The data and code used to obtain the estimates are available at https://www.openicpsr.org/openicpsr/project/217201.

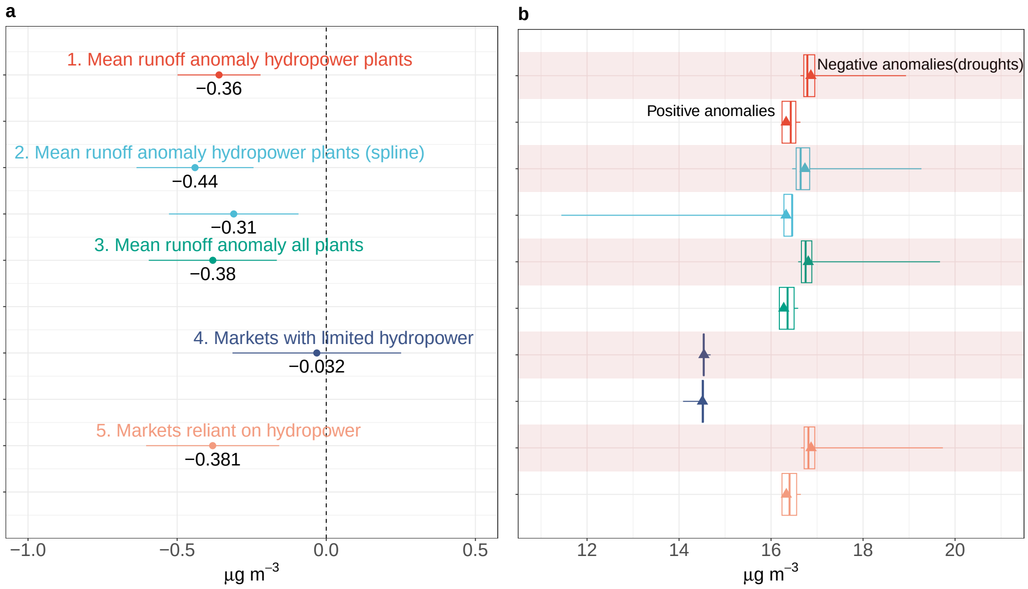

Supplementary Figure 14 Robustness of estimates under alternative definitions of the treatment. a The figure plots point estimates (points) and 95% confidence intervals (error bars) of the β coefficient from five specifications of Equation (1). The CIs are derived from standard errors clustered at the market level. Models 1 to 5 estimate Equation (1), each using the hydrological drought measure listed on top of the coefficient. Negative coefficients imply an increase in pollution for droughts (negative runoff anomalies) and a decrease in pollution for downpours (positive runoff anomalies). Models 1 and 2 use the analysis sample (N = 79,022). Models 3 to 5 use an extended sample that additionally includes Cuba, Guyana, and Trinidad and Tobago (N = 80,355). Markets with limited hydropower are those with less than 20% of generation coming from hydropower, and markets reliant are those with 20% or more. **b** Distribution of implied total $PM_{2.5}$ concentrations, that is, the marginal effect plus the predicted level of $PM_{2.5}$ in the absence of droughts. Box plots indicate median (middle line), 25th, 75th percentile (box), and minimum and maximum (whiskers) as well as mean values (triangles). Source data are provided as a Source Data file (sourcedata.xlsx). The data and code used to obtain the estimates are available at https://www.openicpsr.org/openicpsr/project/217201.

Supplementary Tables

Supplementary Table 1 Descriptive statistics

	Mean	Std. Dev.	Min	Max
Combustion power plants (analysis dataset)				
$PM_{2.5}$	16.60	8.57	2.39	89.42
FHD	0.54	0.30	0.00	1.00
Temperature (°C)	20.47	6.09	-4.74	33.59
Total precipitation(mm/h)	0.21	0.17	0.00	1.24
Relative humidity (%)	77.51	11.87	14.20	93.56
Wind speed (m/s at 10m)	2.77	1.24	0.70	9.21
Wind speed (m/s at 100m)	4.11	1.68	1.07	12.10
Surface pressure (hPa)	966.27	55.68	641.25	1022.56
Heating degree-days (°C days)	74.58	121.99	0.00	459.64
Cooling degree-days (°C days)	145.12	89.87	0.00	308.39
Non-combustion power plants (placebo exercise)	Mean	Std. Dev.	Min	Max
$PM_{2.5}$	12.15	4.97	2.32	74.98
FHD	0.55	0.31	0.00	1.00
Temperature (°C)	19.66	6.16	-6.17	33.73
Total precipitation(mm/h)	0.12	0.14	0.00	1.13
Relative humidity (%)	65.57	19.12	7.03	91.94
Wind speed (m/s at 10m)	3.63	1.44	0.86	8.64
Wind speed (m/s at 100m)	5.01	2.11	1.21	11.95
Surface pressure (hPa)	937.74	87.12	617.20	1021.80
Heating degree-days (°C days)	94.47	131.79	0.00	459.64
Cooling degree-days (°C days)	124.93	95.75	0.00	292.09

This table presents descriptive statistics for key variables in the analysis. The combustion power plant dataset consists of 79,022 observations across 19 markets. The non-combustion power plant dataset, used in placebo exercises, consists of 62,066 observations across 18 markets.

Supplementary Table 2 Climate and earth system models used for projections of runoff anomalies

Institution	Model	Ensemble variant
Australian Community Climate and Earth System Simulator	ACCESS-CM2	r1i1p1f1
Beijing Climate Center Climate System Model	BCC-CSM2-MR	r1i1p1f1
Canadian Earth System Model	CanESM5-CanOE	r1i1p2f1
Community Earth System Model	CESM2	r4i1p1f1
Centro Euro-Mediterraneo sui Cambiamenti Climatici Climate Model	CMCC-CM2-SR5	r1i1p1f1
France Centre National de Recherches Météorologiques	CNRM-CM6-1	r1i1p1f2
France Centre National de Recherches Météorologiques	CNRM-CM6-1-HR	r1i1p1f2
France Centre National de Recherches Météorologiques	CNRM-ESM2-1	r1i1p1f2
European Consortium for Earth System Modeling	EC-Earth3-Veg-LR	r1i1p1f1
Flexible Global Ocean-Atmosphere-Land System Model	FGOALS-g3	r1i1p1f1
Geophysical Fluid Dynamics Laboratory Earth System	GFDL-ESM4	r1i1p1f1
Institute for Numerical Mathematics	INM-CM4-8	r1i1p1f1
Institute for Numerical Mathematics	INM-CM5-0	r1i1p1f1
Institut Pierre-Simon Laplace	IPSL-CM6A-LR	r1i1p1f1
Model for Interdisciplinary Research on Climate	MIROC-ES2L	r1i1p1f2
Model for Interdisciplinary Research on Climate	MIROC6	r1i1p1f1
Max Planck Institute Earth System Model	MPI-ESM1-2-LR	r1i1p1f1
Max Planck Institute Earth System Model	MRI-ESM2-0	r1i1p1f1
Norwegian Earth System Model	NorESM2-LM	r1i1p1f1
Norwegian Earth System Model	NorESM2-MM	r1i1p1f1
Taiwan Earth System Model	TaiESM1	r1i1p1f1
Taiwan Earth System Model	UKESM1-0-LL	r1i1p1f2

We use monthly runoff data from 22 models from CMIP6[5] together with runoff climatologies from IEA[6] to construct projections of runoff anomalies for each watershed in LAC from 2020 to 2059. The choice of models from the CMIP6 is based on their availability given all combinations of Shared Socioeconomic Pathways (SSP) and Representative Concentration Pathways (RCP) scenarios considered in the paper. Each model is detailed with its name and the specific ensemble variant used in our analysis.

Supplementary Methods 1

Alternative statistical models and robustness checks

A key threat to the validity of our research design is the possibility that meteorological factors or changes in electricity demand may confound our estimates of the impact of hydrological droughts on $PM_{2.5}$. While we control for these factors in Equation (1), they enter our econometric model linearly, and we assume that their impact on $PM_{2.5}$ is independent of each other. We conducted several exercises to test that our results are robust to these assumptions.

Supplementary Figure 8 plots point estimates and confidence intervals from these exercises. In the first exercise, we introduce into Equation (1) the meteorological and electricity demand controls in steps. As seen in models 1 to 3, we find a consistent and statistically significant effect of FHD on excess $PM_{2.5}$, albeit with the smallest point estimate on the specification that only includes fixed effects. In the second exercise, we relax the assumption of linearity and additivity by including a far richer set of controls. Specifically, in model 4, we include second-order polynomials of each control variable (each element of the $\mathbf{X}$ vector) and all pairwise interactions (62 additional controls). Analogously, in model 5, we include third-order polynomials of each control variable and all pairwise interactions (494 additional controls). Simply including these controls in Equation (1) and performing the OLS estimation would make the assumption of including all relevant confounds more plausible, but it would come at the cost of lowering the precision of our estimates. To address this tradeoff, we use the more efficient post-double selection method of Belloni et al.[2], which penalizes the models using a two-step LASSO procedure to select a subset of control variables that are important for predicting both $PM_{2.5}$ and FHD. This method allows us to guard against omitted-variables bias by considering these larger sets of controls while using a parsimonious model that produces valid inferential statements. As seen in Fig. 8, models 4 and 5 produce estimates of FHD that are statistically different from zero and whose 95% confidence intervals contain the point estimate from our benchmark specification (model 3). Accordingly, we conclude that our results are robust to the model specification.

Another important concern in our analysis is the potential confounding effect of emissions from wildfires or dust storms. While our primary analysis sample excludes observations with fire emissions within 50 km, we perform additional robustness tests to assess the impact of extending the exclusion radii and excluding dust emission sources. Supplementary Figure 9 presents the results of this sensitivity analysis. It shows the point estimates and 95% confidence intervals of β from Equation (1), derived from standard errors clustered at the market level. Each row corresponds to a different exclusion criterion: the benchmark excludes observations with fire emissions within 50 km, while subsequent models progressively expand the exclusion radius to 75 km and 100 km (models 1, 3, 5). In addition, we test the combined exclusion of observations plausibly affected by fire or dust emissions within these radii (models 2, 4, 6).

The results indicate consistent estimates of β across all specifications, confirming the robustness of our findings to stricter exclusion criteria for plant-month observations potentially affected by fire or dust emissions. While larger exclusion radii result in wider confidence intervals due to reduced sample sizes, the point estimates are of a similar magnitude, demonstrating that the influence of wildfire and dust storm emissions does not significantly alter the relationship between hydrological drought and $PM_{2.5}$ levels.

Next, we test whether the inference of standard errors used to construct confidence intervals is robust to alternative assumptions and methods. Supplementary Figure 10 presents results from these exercises. Model 1 plots the point estimate and confidence interval for the benchmark model. The confidence interval is constructed from standard errors clustered at the market (country) level. We present this type of confidence interval throughout the

paper. Model 2 presents confidence sets derived using the wild cluster bootstrap method[3]. This method provides valuable complementary evidence as it is robust to violations of the standard large-sample assumptions necessary to derive cluster standard errors (e.g., large number clusters, homogeneous size). As the figure highlights, this more robust method produces a wider confidence set, but reassuringly, it shows that FHD remains statistically different from zero at conventional levels.

Another assumption necessary for clustered standard errors to be valid is that observations in different clusters are independent of each other. While this assumption is likely to hold in our setting, given that our clustering units (markets) are much larger than our observational units (plants), it is still possible for plants located on opposite sides of a cluster boundary to be affected by a correlated and unobserved shock. To account for this possibility and the resulting greater likelihood of type 1 errors, in models 3 and 4, we move away from using non-overlapping clusters and compute Conley errors using the methodology of Colella et al.[4]. Following their recommendations, we assume a binary covariance matrix, fully account for temporal dependence by allowing the lag cutoff to equal the length of our panel, and present results with a narrow and wide distance cutoff (50 km and 5,000 km). The wide distance cutoff exceeds the length of Chile (the longest country in our sample). As seen in Fig. 10, the confidence intervals derived from Conley errors (models 3 and 4) are much narrower than those of our benchmark (model 1), confirming in all cases that the estimate of FHD is statistically different from zero. These findings highlight that the confidence intervals used throughout the paper do a good job of quantifying the uncertainty associated with estimating FHD and that our conclusions are robust to alternative assumptions and methods for estimating standard errors.

We now turn to assess the robustness of our results to alternative ways of measuring $PM_{2.5}$ concentrations. Supplementary Figure 11 presents results from several sensitivity exercises. In panel a, model 1 reports the benchmark results, where the outcome is the mean monthly $PM_{2.5}$ concentration within 50 km of combustion power plants. Model 2 uses the same approach but focuses on mean monthly $PM_{2.5}$ concentrations within 10 km of combustion power plants. As anticipated, the analysis produces similar coefficients, with slightly higher implied concentrations in the model that uses measurements within a 10 km radius (panel b).

Throughout the paper, we rely on modeled $PM_{2.5}$ concentrations from Van Donkelaar et al.[7], which combines satellite data, chemical transport models, and ground-based monitors data to produce global, high-resolution estimates of $PM_{2.5}$ concentrations. For simplicity, we refer to this dataset as satellite-derived $PM_{2.5}$. This dataset is well-suited for our analysis because it offers consistent, long-term monthly coverage across the entire region. However, a key concern is the uncertainty inherent in satellite-derived $PM_{2.5}$ data and its potential impact on our results.

To address this concern, we conducted a comprehensive review of ground-level monitoring data in the region and were able to obtain hourly $PM_{2.5}$ measurements from monitoring stations operating between 2000 and 2020 in Argentina, Brazil, Colombia, Chile, Ecuador, Peru, and Mexico[8–15]. Using this dataset, we calculated monitor-level mean monthly $PM_{2.5}$ concentrations. For each plant-month observation, we then assigned the $PM_{2.5}$ information from the nearest ground monitoring station within 50 km. Due to the sparse network of ground monitors in the region, the resulting sample is smaller (N = 1,797). In panel a, we present the results for models 3 and 4, estimated using Equation (1) with this sample. Model 3 relies on satellite-derived $PM_{2.5}$, while model 4 uses ground-monitor $PM_{2.5}$. Despite the smaller sample size, both models reveal a consistent and statistically significant effect of FHD on excess $PM_{2.5}$. The confidence intervals indicate that the effect sizes are comparable to each other and align with the benchmark finding (model 1). The larger point

estimate and implied concentrations in model 4 (panels a and b) are likely due to a substantial proportion of ground-level monitors being located in close proximity to combustion power plants, with a median distance of 7 km.

Panels c and d further explore the relationship between satellite-derived and ground-monitor $PM_{2.5}$. Panel c shows a strong positive correlation ($\rho = 0.82$) between these two measures for the ground-monitor subsample, though some nonlinearity is observed at higher ground-monitor $PM_{2.5}$ values. This nonlinearity is likely due to the comparison between satellite-derived mean concentrations within a 50 km radius of the plant and ground-monitor data collected at a single point within that radius. Since a large fraction of ground monitors are located close to plants, they tend to capture higher localized concentrations, which may contribute to the observed deviation from linearity. Panel d compares the distributions of $PM_{2.5}$ from the two datasets, revealing broadly similar patterns albeit with slightly higher average concentrations in the ground-monitor data. These results suggest that satellite-derived $PM_{2.5}$ provides a reliable basis for our analysis, aligning well with ground-monitor data despite differences in spatial coverage and measurement methods.

Next we turn our attention to testing whether our results are robust to using alternative variable definitions. We begin with the definition of hydrological drought used to compute the FHD variable. Throughout the paper, we have defined that a hydropower plant experiences a hydrological drought when we observe an average negative runoff anomaly over the past three months. To assess the importance of this definition as described in the data section, we compute alternative versions of the FHD variable where we define drought as observing average negative runoff anomalies over averaging windows of length 1, 6, 9, and 12 months. Supplementary Figure 12 reports the results from separately estimating Equation (1) using each version of the FHD. Panel a presents point estimates and confidence intervals. Panel b plots the implied total $PM_{2.5}$ concentrations ($PM_{2.5}$ concentrations in the absence of droughts plus the marginal effect). The figure shows that estimates of the FHD are similar for all definitions, albeit slightly larger when using shorter averaging windows. This finding is important because it highlights that even short-run droughts can considerably increase $PM_{2.5}$ concentrations in LAC. This finding is consistent with the widespread presence of small run-of-river hydropower plants in the region, for which even short-run changes in water availability imply reduced generation capacity.

We also test and find similar results using alternative ways of aggregating our watershed measure identifying hydropower plants affected by drought to the market level. The first type of exercise sequentially estimates Equation (1) using alternative versions of our preferred market-level measure, the fraction of hydropower generation affected by drought (FHD). The results from these exercises, shown in Fig. 13a,b, are analogous to those of the previous figure. Model 1 reports the estimate from the benchmark. Model 2 uses an alternative version of FHD that uses an arithmetic average instead of weighting by generation capacity. Model 3 computes the FHD as a fraction of overall generation instead of a fraction of hydropower generation. Model 4 allows for regional markets and computes the FHD, assuming that markets with small amounts of cross-border trade are fully integrated. Specifically, we compute the FDH considering that the following countries are part of larger regional markets. Market 1: Colombia, Ecuador, and Venezuela. Market 2: Brazil, Uruguay, Paraguay. Market 3: Central America. Model 5 performs the same calculation of the FHD as model 4 but uses an arithmetic average instead of weighting by generation capacity. Models 6 and 7 test the impact of FHD when we use more severe definitions of drought. In these cases, we compute the FDH either assuming that droughts occur when mean runoff anomalies are less than minus one standard deviation or when they are less than the median of negative anomalies. As seen in Fig. 13, we find very similar results across models. While in a, some versions of the FHD lead to re-scaled and, in some cases, larger point estimates, b shows that the implied total $PM_{2.5}$ concentrations are nearly identical.

The second type of exercise also aims to assess the robustness of the results to alternative market-level measures of hydrological drought. However, it focuses on measures that directly gauge the intensity of droughts. Supplementary Figure 14 presents results from these exercises. Panels a and b are analogous to those of the previous figure. We begin by computing the mean runoff anomaly among hydropower plants for each market. For ease of presentation, we convert this measure to standard deviation units and estimate Equation (1) using this variable in place of HD. We are particularly interested in negative runoff anomalies as they represent periods when water availability is below normal. As seen in a, model 1, a one-standard-deviation negative runoff anomaly leads to an increase of 0.36 µg m^{-3}. As shown in b, this estimate also implies that an average negative runoff anomaly leads to $PM_{2.5}$ concentration of 16.86 µg m^{-3}. This concentration level is very similar to the level implied by our benchmark specification estimate (16.59 µg m^{-3} at the average FHD). To test whether negative and positive runoff anomalies have symmetric effects on $PM_{2.5}$, in model 2, we re-estimate Equation (1) but allow for a spline with a kink at zero. That is, we allow the slope coefficient to differ for positive and negative runoff anomalies. The estimates in a, while of a similar magnitude, indicate that one-standard-deviation negative runoff anomaly (drought) leads to a larger increase (0.44 µg m^{-3}) in $PM_{2.5}$ than the decrease in $PM_{2.5}$ that one-standard-deviation positive runoff anomalies would generate (-0.31 µg m^{-3}). This asymmetric result is important because it highlights that the externality created by droughts cannot be offset by downpours. The smaller effect of positive runoff anomalies is consistent with the idea that infrastructure and operational limitations may restrict the ability of hydropower plants to fully harness downpour periods to increase generation. Next, in models 3 to 5, we compute the mean runoff anomaly among all power plants for each market. This change allows us to incorporate into the analysis three additional markets (Cuba, Guyana, and Trinidad and Tobago) that do not have hydropower generation in our sample. In model 3, we estimate Equation (1) using this new measure and this slightly larger sample. In model 4, we restrict the sample to markets with limited hydropower capacity (less than 20% of overall generation). Finally, in model 5, we restrict the sample to markets reliant on hydropower (20% or more of overall generation). The figure shows that hydrological droughts affecting markets reliant on hydropower drive the increase in $PM_{2.5}$. Importantly, consistent with our mechanism, we also fail to find evidence of hydrological droughts leading to excess $PM_{2.5}$ in markets where hydropower plays a limited role in electricity generation.

Supplementary Methods 2

Alternative calculation of lives lost and losses due to excess $PM_{2.5}$

To compute plant-month-level premature deaths, we combine our benchmark estimate of drought-induced excess $PM_{2.5}$ (Fig. 1a) with several well-established concentration-response functions (CRFs) and estimates of baseline deaths. The approach varies according to the CRF source.

Liu et al. (2019): For each plant-month, we use the reported change in all-cause mortality per 1µg m^{-3} increase in $PM_{2.5}$ (0.129% for Mexico, 0.027% for Chile). We multiply this percentage by the estimated excess $PM_{2.5}$ and daily baseline deaths. Daily baseline deaths are derived by multiplying annual mortality rates by the exposed population, then dividing by 365. We scale the resulting premature deaths to a monthly total by multiplying by 30. This CRF applies to all age groups. Mortality rates are country and year-specific. These rates are sourced from the World Bank[16].

World Health Organization (2020) [AirQ+ Log-linear]: We apply the WHO AirQ+ log-linear model with a beta coefficient ($\beta = 0.0077$) and a threshold of 5 µg m^{-3} to compute relative risk ratios based on observed and counterfactual (no drought) excess $PM_{2.5}$. These

relative risk ratios are then used to compute the fraction of deaths attributable to drought-induced excess $PM_{2.5}$. Then, for each plant month observation, we multiply this fraction by monthly baseline deaths, obtained by multiplying annual mortality rates by the exposed population and dividing by 12. Because this CRF pertains to individuals aged 25 years or older, we further scale baseline deaths by the fraction of the population older than 25. The demographic data is sourced from the UN[17].

Global Exposure Mortality Model (GEMM) [based on Burnett et al. (2018)]: The GEMM model accounts for non-linear and saturating concentration-response relationships. The model uses parameters $\theta = 0.143$, $\alpha = 1.6$, $\mu = 15.5$, $\pi = 36.8$ and a threshold of 2.4 µg m^{-3}. We compute relative risks from observed and counterfactual excess $PM_{2.5}$ using this model. We then derive the attributable fraction of deaths and multiply it by the monthly baseline deaths calculated as before. Consistent with GEMM, the exposed population includes adults aged 25 and older.

U.S. Environmental Protection Agency [based on Pope et al. (2019)]: For each plant-month, we compute the change in mortality risk attributable to drought-induced excess $PM_{2.5}$ using an exponential decay function with $\beta = 0.01133$. We then multiply this function with monthly baseline deaths, which are calculated analogously to the other methods. In line with the CRF, this calculation focuses on individuals aged 30 years or older.

In these exercises, we also account for the uncertainty in our estimate of drought-induced excess $PM_{2.5}$ by performing simulations where we take 1,000 draws from a normal distribution centered at our point estimate (1.55) and with a standard deviation equal to the standard error (0.31). To construct Fig. 1, we aggregate the resulting dataset to the LAC-year level. For Fig. 3, we monetize the lives lost by multiplying the previously derived dataset by country and year-specific estimates of the value of a statistical life and then aggregate the results to the LAC-year level.

Supplementary References

1. International Energy Agency. *Latin America Energy Outlook 2023* (IEA, Paris, 2023).

2. Belloni, A., Chernozhukov, V., Hansen, C. & Kozbur, D. Inference in high-dimensional panel models with an application to gun control. *Journal of Business & Economic Statistics* **34**, 590–605 (2016).

3. Roodman, D., Nielsen, M. Ø., MacKinnon, J. G. & Webb, M. D. Fast and wild: Bootstrap inference in stata using boottest. *The Stata Journal* **19**, 4–60 (2019).

4. Colella, F., Lalive, R., Sakalli, S. O. & Thoenig, M. acreg: Arbitrary correlation regression. *The Stata Journal* **23**, 119–147 (2023).

5. Copernicus Climate Change Service. CMIP6 climate projections. *Climate Data Store (C3S)* (2021). DOI: 10.24381/cds.c866074c .

6. International Energy Agency. Weather for Energy Tracker database, V1222. *IEA* (2022). http://weatherforenergydata.iea.org/.

7. Van Donkelaar, A. *et al.* Monthly global estimates of fine particulate matter and their uncertainty, v5gl03. *Atmospheric Composition Analysis Group Washington University in St. Louis* (2021). https://sites.wustl.edu/acag/datasets/surface-pm2-5/.

8. Datos Argentina. Calidad del Aire, V2024. *Datos Medio Ambiente* (2024). https://datos.gob.ar/dataset/ambiente-calidad-aire.

9. SPARTAN Network. Buenos Aires, Argentina (ARCB), V2024. *SPARTAN data repository* (2024). https://www.spartan-network.org/buenos-aires-argentina.

10. Instituto de Energia e Meio Ambiente. Qualidade do Ar, V2024. *Platforma da Qualidade do Ar* (2024). https://energiaeambiente.org.br/qualidadedoar/en/.

11. Instituto de Hidrología, Meteorología y Estudios Ambientales. Calidad del Aire, V2024. *Sistema de Informacíon sobre Calidad del Aire* (2024). http://www.siac.gov.co/sisaire.

12. Ministerio del Medio Ambiente Chile. Información Historica, V2024. *Sistema de Información Nacional de Calidad del Aire* (2024). https://sinca.mma.gob.cl.

13. Red Metropolitana de Monitoreo Atmosférico de Quito. Datos de Particulas Menores a 2.5 micrometros, V2024. *Datos Historicos REMMAQ* (2024). https://datosambiente.quito.gob.ec.

14. U.S. Department of State. Historical Data Lima, V2024. *AirNow* (2024). https://www.airnow.gov/international/us-embassies-and-consulates/.

15. Instituto Nacional de Ecología y Cambio Climático. Datos Crudos, V2024. *Sistema Nacional de Información de la Calidad del Aire* (2024). https://sinaica.inecc.gob.mx/index.php.

16. World Bank. Death rate, crude, v2023. *World Development Indicators* (2023). https://databank.worldbank.org/source/world-development-indicators.

17. United Nations. Total population, both sexes combined, v2024. *UN Data* (2023). https://data.un.org/Data.aspx?q=population&d=PopDiv&f=variableID%3a12.
