# Supplementary material for: Droughts worsen air quality and health by shifting power generation
Source: Nat Commun. 2025 May 22;16:4774. doi: 10.1038/s41467-025-60090-z (PMC12098700; doi:10.1038/s41467-025-60090-z)
Supplement: Supplementary file 2 — Reporting Summary [file 41467_2025_60090_MOESM2_ESM.pdf]

nature portfolio

Corresponding author(s): Mathilda Eriksson and Alejandro del Valle

Last updated by author(s): Apr 23, 2025

Reporting Summary

Nature Portfolio wishes to improve the reproducibility of the work that we publish. This form provides structure for consistency and transparency in reporting. For further information on Nature Portfolio policies, see our Editorial Policies and the Editorial Policy Checklist.

Statistics

For all statistical analyses, confirm that the following items are present in the figure legend, table legend, main text, or Methods section.

n/a	Confirmed	
☐	☒	The exact sample size (*n*) for each experimental group/condition, given as a discrete number and unit of measurement
☐	☒	A statement on whether measurements were taken from distinct samples or whether the same sample was measured repeatedly
☐	☒	The statistical test(s) used AND whether they are one- or two-sided<br>*Only common tests should be described solely by name; describe more complex techniques in the Methods section.*
☐	☒	A description of all covariates tested
☒	☐	A description of any assumptions or corrections, such as tests of normality and adjustment for multiple comparisons
☐	☒	A full description of the statistical parameters including central tendency (e.g. means) or other basic estimates (e.g. regression coefficient) AND variation (e.g. standard deviation) or associated estimates of uncertainty (e.g. confidence intervals)
☐	☒	For null hypothesis testing, the test statistic (e.g. *F*, *t*, *r*) with confidence intervals, effect sizes, degrees of freedom and *P* value noted<br>*Give P values as exact values whenever suitable.*
☒	☐	For Bayesian analysis, information on the choice of priors and Markov chain Monte Carlo settings
☒	☐	For hierarchical and complex designs, identification of the appropriate level for tests and full reporting of outcomes
☒	☐	Estimates of effect sizes (e.g. Cohen's *d*, Pearson's *r*), indicating how they were calculated

Our web collection on statistics for biologists contains articles on many of the points above.

Software and code

Policy information about availability of computer code

Data collection	No software was used for data collection
Data analysis	Data analysis was performed with Stata MP v17.0, and R v4.3.2. In all cases, we only used publicly accessible functions and packages. The code used in the study is provided in the paper's openICPSR repository https://www.openicpsr.org/openicpsr/project/217201.

For manuscripts utilizing custom algorithms or software that are central to the research but not yet described in published literature, software must be made available to editors and reviewers. We strongly encourage code deposition in a community repository (e.g. GitHub). See the Nature Portfolio guidelines for submitting code & software for further information.

Data

Policy information about availability of data

All manuscripts must include a data availability statement. This statement should provide the following information, where applicable:

- Accession codes, unique identifiers, or web links for publicly available datasets
- A description of any restrictions on data availability
- For clinical datasets or third party data, please ensure that the statement adheres to our policy

All data used in the paper was collected from publicly available sources. These datasets include data on power plant characteristics, runoff anomalies, pm 2.5 concentrations, meteorological conditions, wildfire emissions, dust emission sources, downscaled demographic information, climate, demographic and growth projections, and downscaled Human Development Index information. All datasets used are cited in the paper. There are no availability restrictions. The datasets

generated and analyzed during the current study are available at the papers openICPSR repository (https://www.openicpsr.org/openicpsr/project/217201). Source Data are provided with this paper.

Research involving human participants, their data, or biological material

Policy information about studies with human participants or human data. See also policy information about sex, gender (identity/presentation), and sexual orientation and race, ethnicity and racism.

Reporting on sex and gender	Our study does not involve human participants, their data, or biological material. We analyze publicly available environmental and operational data on power plants, which do not contain any individual-level human subject information
Reporting on race, ethnicity, or other socially relevant groupings	Our study does not involve individual-level data and does not collect or analyze information on race, ethnicity, or other socially relevant groupings. All data used are environmental and operational in nature, aggregated at the level of power plants and geographic regions.
Population characteristics	See above.
Recruitment	No participant were recruited.
Ethics oversight	No ethics oversight was requested for this research because our analysis uses exclusively publicly available, non-sensitive data. Specifically, our study relies on:<br><br>Power plant data from public databases<br>Environmental monitoring data from remote sensing<br>Aggregated population data from census datasets and other public sources<br><br>All data used in this study is completely anonymized and contains no personally identifiable information. The population data used for our projections is aggregated at the census block or larger geographic level, ensuring that no individual privacy concerns arise from the analysis. This research does not involve human subjects, private information, or sensitive data requiring IRB review.

Note that full information on the approval of the study protocol must also be provided in the manuscript.

Field-specific reporting

Please select the one below that is the best fit for your research. If you are not sure, read the appropriate sections before making your selection.

☐ Life sciences ☒ Behavioural & social sciences ☐ Ecological, evolutionary & environmental sciences

For a reference copy of the document with all sections, see nature.com/documents/nr-reporting-summary-flat.pdf

Behavioural & social sciences study design

All studies must disclose on these points even when the disclosure is negative.

Study description	Study Description: This study investigates the impact of hydrological droughts on air quality in Latin America and the Caribbean (LAC), focusing on the increase in fine particulate matter (PM2.5) levels due to shifts in power generation from hydropower to combustion power plants. The study spans 21 years (2000-2020) and utilizes a plant-month-level panel dataset that includes data on air quality, meteorological conditions, and hydrological data.<br><br>Quantitative Data and Design Structure: The study employs a quantitative quasi-experimental design with fixed effect methods to estimate the causal impact of drought-induced shifts in power generation on PM2.5 concentrations. The design structure includes the following elements:<br><br>The primary measure of hydrological drought is the market-level fraction of hydropower generation capacity affected by drought (FHD), calculated from hydropower watershed-level measures of runoff anomalies. Supporting evidence for the shift in power generation as the primary mechanism is provided by ruling out alternative mechanisms and by examining interactions with plant size and fuel type.<br><br>Design Structure: The study follows a regression analysis design, examining the relationship between FHD and PM 2.5 concentrations near combustion power plants. The analysis controls for meteorological variables, electricity demand, seasonal trends, unobserved time-invariant characteristics of plants, and unobserved time-varying characteristics common to all units in the region. We also provide placebo tests to rule out that the results are not driven by confounders such as air pollution from wildfires or dust storms.<br><br>The study also uses well established concentration response functions to report the effect of excess PM 2.5 in terms of premature deaths, and existing estimates of the value of statistical life to report losses in year 2019 US dollars. The paper also uses climate runoff projections from the CMPI6, demographic and economic projections from the SSP-RCPs, and energy projection from the IEA to project damages up to 2059.
Research sample	The research sample for this study is monthly observations for combustion power plants (N=1,835) across Latin America and the Caribbean over a 21 year period (2000–2020). This dataset includes detailed monthly observations on PM2.5 concentrations,

	meteorological and hydrological conditions, and power plant characteristics. The dataset does not include individual-level human subject data, so demographic information such as sex is not applicable.<br><br>The analysis dataset was constructed using several public sources, selected to ensure the broadest possible geographical coverage. The source datasets and the process for building the analysis dataset are described in detail in the Methods section.
Sampling strategy	No sampling or power calculation were performed. All publicly available data on power plants in LAC was used.
Data collection	The analysis dataset was assemble from several publicly available datasets cited in the paper.
Timing	The data for this project was collected from publicly available sources between March and October 2023. The additional data collected for the resubmission was collected between October 2024 and January 2025.
Data exclusions	Our analysis sample excludes plant-month observations where carbon emissions from wildfires are observed within 50km of power plants. We do so to mitigate the confounding effect of wildfires. The choice of sample was made before data collection, and it followed the strategy used by previous papers. As requested by reviewers in the supplementary information, we also provide additional robustness checks where we exclude plant-month observations plausibly affected by wildfires or dust emissions within a 75 and 100-km radius.
Non-participation	This study does not involve human participants. All data are observational and derived from publicly available sources on power plant and environmental conditions. Therefore, there was no recruitment, consent, or risk of non-participation.
Randomization	This is an observational study, no randomization or assignment to experimental groups was conducted. Instead, we rely on quasi-experimental methods, including fixed-effects regression models and extensive covariate controls. Please see methods for a detailed discussion.

Reporting for specific materials, systems and methods

We require information from authors about some types of materials, experimental systems and methods used in many studies. Here, indicate whether each material, system or method listed is relevant to your study. If you are not sure if a list item applies to your research, read the appropriate section before selecting a response.

Materials & experimental systems

n/a	Involved in the study	
☒	☐	Antibodies
☒	☐	Eukaryotic cell lines
☒	☐	Palaeontology and archaeology
☒	☐	Animals and other organisms
☒	☐	Clinical data
☒	☐	Dual use research of concern
☒	☐	Plants

Methods

n/a	Involved in the study	
☒	☐	ChIP-seq
☒	☐	Flow cytometry
☒	☐	MRI-based neuroimaging

Plants

Seed stocks	Not applicable
Novel plant genotypes	Not applicable
Authentication	Not applicable
